# Inflammatory monocyte response due to altered wall shear stress in an isolated femoral artery model

**DOI:** 10.14440/jbm.2019.274

**Published:** 2019-02-20

**Authors:** Aparna A. Kadam, Robert P. Gersch, Todd K. Rosengart, Mary D. Frame

**Affiliations:** 1 Department of Biomedical Engineering, Physiology and Biophysics, Stony Brook University, Stony Brook, NY 11794-5281, USA; 2 Department of Surgery, Stony Brook University, Stony Brook, NY 11794-5281, USA; 3 Department of Surgery, Baylor College of Medicine, Houston, TX 77030, USA

**Keywords:** arteriogenesis, femoral artery, inflammation, monocyte, wall shear stress

## Abstract

Arteriogenesis (collateral formation) is accompanied by a pro-inflammatory state that may be related to the wall shear stress (WSS) within the neo-collateral vessels. Examining the pro-inflammatory component *in situ* or *in vivo* is complex. In an *ex vivo* mouse femoral artery perfusion model, we examined the effect of wall shear stress on pro-arteriogenic inflammatory markers and monocyte adhesion. In a femoral artery model with defined pulsatile flow, WSS was controlled (at physiological stress, 1.4×, and 2× physiological stress) during a 24 h perfusion before gene expression levels and monocyte adhesion were assessed. Significant upregulation of expression was found for the cytokine TNFα, adhesion molecule ICAM-1, growth factor TGFβ, and the transcription factor Egr-1 at varying levels of increased WSS compared to physiological control. Further, trends toward upregulation were found for FGF-2, the cytokine MCP-1 and adhesion molecules VCAM-1 and P-selectin with increased WSS. Finally, monocytes adhesion increased in response to increased WSS. We have developed a murine femoral artery model for studying changes in WSS *ex vivo* and show that the artery responds by upregulating inflammatory cytokines, adhesion molecules and growth factors consistent with previous *in vivo* findings.

## INTRODUCTION

In the adult, vascular growth occurs *via* either angiogenesis or arteriogenesis. Angiogenesis is characterized by the hypoxia induced, VEGF driven, de novo sprouting of capillaries that are formed from endothelial cells. These initial capillaries then elongate and sprout to generate capillary networks [1]. Arteriogenesis (collateralization) is vascular growth stemming from the remodeling of pre-existing arterioles with multiple triggers and signaling pathways that remain to be defined [2]. Collaterals form primarily *via* the process of arteriogenesis. The processes involve some of the same growth factors, but the conditions that initiate each process distinguish the two. The triggers for angiogenesis are largely accepted to be ischemia and hypoxia; the triggers for arteriogenesis are still the subject of much debate and are less defined. Mechanical effects due to increased blood flow comprise the leading hypotheses regarding triggers for collateral growth in these vascular networks [3-5].

In cases of vascular occlusion, the ability to elicit a neovascular response (arteriogenesis) to form collateral vessels can protect blood flow by providing an alternate stable route for blood flow to occluded regions. Understanding the mechanical factors and mechanisms that trigger and control arteriogenesis may allow for targeted treatment to mediate the formation of these collateral networks when and where necessary. *In vivo* studies of arterial occlusions using the femoral artery excision (FAE) or ligation models have provided valuable information to the field of study [6,7]. However, specific conditions at particular vascular sites are difficult to control and any chemical or surgical interventions inevitably cause systemic changes that cannot be controlled. By using an *ex vivo* model we will be able to control the level of inflammation chemically or mechanically with TNFα treatment or by increasing the flow rate through the artery, respectively.

Our hypothesis is that increasing wall shear stress (WSS) leads to increased growth factor and adhesion molecule expression as well as leukocyte recruitment and adhesion, specifically monocytes which are integral to collateral formation [7-9]. The monocytes themselves produce MMP’s that are able to break down extracellular matrix on their progression through the vessel wall. It has specifically been shown that the stimulation of arteriogenesis with monocyte chemoattractant protein 1 (MCP-1) augments the expression of several MMP’s (MMP-1, 2, 3, 9) [10]. The MMPs aid in the breakdown of the extracellular matrix and skeletal muscle cells to create more space for the growth of the vessel. Finally, the recruited monocytes produce TNFα and FGF-2 that further aid in the proliferation of endothelial and smooth muscle cells [3].

Here we aim to develop and characterize an *ex vivo* femoral artery system for studying mechanical triggers leading to pro vasculogenic inflammatory conditions and to subsequently use this system to study pathophysiology of arterial inflammatory conditions. After characterization, this study aims to address: (1) the elucidation of the inflammatory response in a femoral artery due to induced increase in WSS in an *ex vivo* femoral artery bioassay chamber, and (2) the study of subsequent downstream monocyte adhesion resulting from the inflammatory response induced by the increase in WSS.

## MATERIALS AND METHODS

Femoral arteries were obtained through a tissue sharing program at Stony Brook University. An *ex vivo* system was designed to sustain perfused femoral arteries for 24 h. The chamber design is comparable to previous *ex vivo* chambers, but designed to the specific needs of this study [11,12].

### Chamber design and implementation

The chamber bottom was constructed from a glass slide with a mold of Sylgard [11] containing two lengthwise ports to secure the cannulated vessel and a passive drain for excess media. Femoral arteries were cannulated with pulled PE50 tubing, stretched to its *in vivo* length (measured pre-extraction), and sutured in place with 7-0 silk sutures. Each vessel was then perfused with media consisting of Dulbecco’s Modified Eagle Medium (DMEM from Gibco) supplemented with 10% fetal bovine serum (FBS) [13,14]. Perfusion media flow was regulated by a pulsatile pump (Watson Marlow 403U/VM2) and was not recirculated. The assay chamber was maintained at 37°C and 5% CO_2_ under sterile conditions (See **Fig. S1**).

### Perfusion media

The osmolality of DMEM with 10% FBS is 300 mosmol/kg which is comparable to the average plasma osmolality reported in male C57BL/6 mice of 315 ± 6 mosmol/kg [15]. The resulting viscosity is *η*_DMEM_ = 8.9 × 10^-4^ Pa·s, which is lower that the viscosity of blood which is *η*_blood_ = 4.0 × 10^-3^ Pa·s. This disparity was accounted for in the flow rate calculations.

### Pulsatile pump characterization

The pulsatility of the pump was characterized using a pressure transducer and a Matlab program designed to visualize data as a waveform (data not shown). Pulsatility was defined by the given equation *y* = 4.99*x*–2.45. Specifically; revolutions per minute (RPM) = 21.5 (physiological) = 104.92 beats/min, comparable to a sedated mouse. RPM = 27.8 (1.4 × physiological) = 136.38 beats/min and RPM = 37.1 (2 × physiological) = 182.83 beats/min imparted the appropriate WSS (**Table 1**). The shape of the waveform as well as the maximum pressure (80 mmHg) stayed constant as the rate is increased.

### Flow rate calculation & induced wall shear stress

The flow rate to maintain physiological WSS (pWSS) in the excised vessel was calculated using the peak flow rate in the iliac branch of an average C57BL/6 mouse (*Q*_max_ = 0.12 ml/min) [16], coupled with the equation for WSS (1). Specifically,
(Eqn 1)
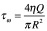


*τ*_ω_ = WSS, *η* = viscosity, *Q* = flow rate, *R* = internal radius of the vessel, and *D* = 2R. Given that *Q*_invivo_ = 0.12 ml/min for the iliac artery *in vivo*, *η*_blood_ = 4.0 × 10^-3^ Pa·s (0.03 Poise), and *D*_femoral_ = 0.20 mm [16], the peak *τ*_ω_ in the femoral artery of an average mouse is 10.18 Pa (101.8 dynes/cm^2^). To determine the peak flow rate required to maintain this shear in the *ex vivo* model: *Q*_exvivo_ where *τ*_ω_ = 10.18 Pa and *η*_DMEM_ = 8.9 × 10^-4^ Pa·s was used. Therefore: *Q*_exvivo_ = 0.53 ml/min [13] (**Table 2**). In order to model the appropriate WSS *ex vivo*, the system needs to compensate for the lower density of perfusion media. To do this, this model will use an increased flow rate as indicated.

Max and average pressure were empirically measured by the pressure transducer at each RPM setting and calculated using the time-averaged method. Physiological peak WSS determined as *τ*_ω_ = 10.18 Pa, medium peak WSS was assumed to be 1.4 × physiological = 14.26 Pa, and high peak WSS was 2 × pWSS = 20.37 Pa. Inducing these peak WSS values were accomplished by changing the flow rate of the perfusate which corresponds to setting average flow rates of 0.4319, 0.5682 and 0.7727 ml/min respectively (**Table 1**).

### Femoral artery and branch isolation

**Figure 1.**
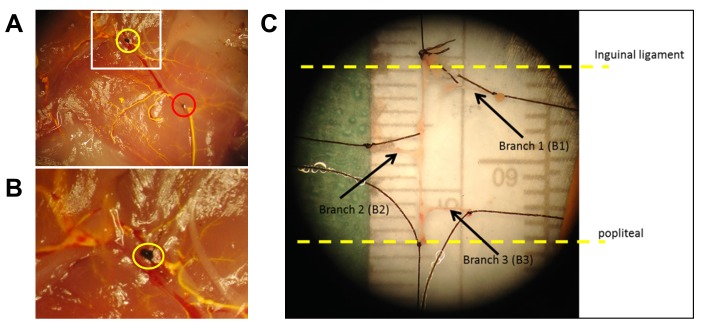
Representative excision site and femoral artery. **A.** Representative excision site after perfusion of yellow contrast agent to visualize the arterial network. This image indicates the proximal end of the artery excised at the inguinal ligament (hip, yellow circle) and the distal end by the popliteal branch (knee, red circle). **B.** High magnification image of the proximal excision site illustrates the femoral vein downstream of the suture, but not the artery. **C.** Once the main artery is ligated and excised, three distinct branches (B1, B2, and B3) are also excised bluntly. After cannulation, the effective average length of the femoral artery is 0.85 cm.

Male C57BL/6 mice aged 10–14 weeks were anesthetized with 5% isoflurane and euthanized by cervical dislocation. The *in vivo* length of the artery was recorded from the inguinal ligament to the popliteal artery bifurcation. The three standard branches of the femoral artery were visualized, ligated, and excised (**Fig. 1**). The femoral artery was cannulated at the inguinal ligament and flushed with saline. Femoral arteries were then split into 4 groups: (1) (*in vivo*) excised and immediately placed in TRIzol for RNA analysis (*n* = 9); (2) (no shear) secured at physiological length in growth media and incubated with no perfusion for 24 h (*n* = 5); (3) pWSS (*n* = 7), 1.4× pWSS (*n* = 7), and 2× pWSS (*n* = 7) cannulated below the popliteal artery branch and perfused as described; or (4) TNFα positive control (*n* = 7) which were perfused at pWSS and supplemented with 1 µg/ml TNFα. The vessels were perfused for 24 h and then analyzed for the inflammatory markers or monocyte adhesion. Vessel prep and perfusion did not appear to result in visible structural damage (**Fig. S2**).

### RNA isolation

Messenger ribonucleic acid (mRNA) was isolated using TRIzol (Ambion of Life Technologies) using the manufacturer’s recommended protocol. Briefly, samples were homogenized prior to chloroform separation. RNA was precipitated using Isopropyl alcohol (IPA) and washed with 75% Ethyl alcohol (EtOH) prior to resuspension in RNase free H_2_O. mRNA concentration was determined using a spectrophotometer and then samples were aliquoted and stored at –80°C.

### Quantitative reverse transcriptase PCR

Sybr-green one-step real time kit (Qiagen) was used to analyze expression of MCP-1, TNFα, P-selectin, vascular cell adhesion molecule 1 (VCAM-1), intercellular adhesion molecule 1 (ICAM-1), Ly6c, FGF-2, TGFβ, VEGF, Egr-1 and Glyceraldehyde 3-phosphate dehydrogenase (GAPDH) expression using manufactures recommended protocols and primers designed using the online Primer3 program. Results were normalized to the housekeeper GAPDH and quantified using the 2^(- ΔΔCT)^ method [17]. Expression values were further normalized to *in vivo* control and reported as average fold change ± standard error.

### Negative mouse monocyte enrichment and activation

The EasySep Monocyte Enrichment Kit (StemCell) was used for this process following manufacturer’s recommended protocols. Briefly, bone marrow was harvested from both femurs of a C57/B6 mouse using a total of 4 ml of “recommended medium” (Phosphate buffered saline) + 2% FBS. Cells were pelleted and a negative selection biotinylated monocyte enrichment antibody cocktail was used. Samples were washed and incubated with a tetrameric antibody complex and magnetic dextran iron particles. An EasySep magnet was then used to separate the monocytes. Manufacturer’s state greater than 85% resulting population is comprised of monocytes after concentration.

Monocytes were exposed to 50 ng/ml of TNFα for 30 min to activate them and labelled with Calcein-AM, washed and immediately used for static incubation studies.

### Static monocyte adhesion

After 24 h at pWSS, 1.4× pWSS, and 2× pWSS, vessels were flayed and statically incubated them with activated monocytes for 1 h (*n* = 4 femoral arteries/group). The vessels were then washed, fixed, and imaged for adherent cells. Vessels were fixed and mounted with DAPI to quantify monocyte adhesion.

### Statistical analysis

All data is represented as fold change over *in vivo* levels and analyzed with respect to physiological peak WSS conditions. Welsh’s corrections were used to account for unequal variances and multiple *t*-tests were used to assess differences between groups. Differences were considered significant for *P* < 0.05.

## RESULTS

### Baseline wall shear stress RNA analysis

**Figure 2.**
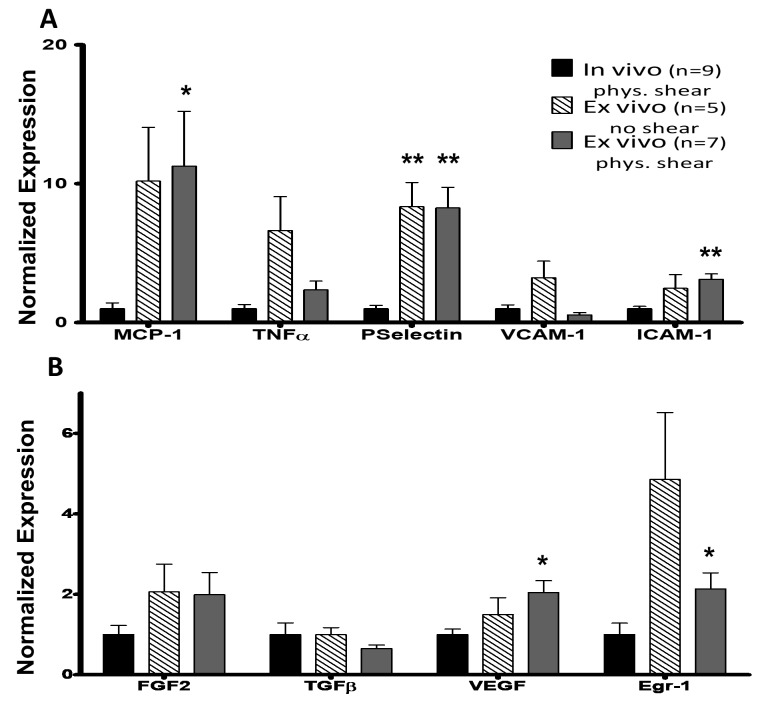
Cytokine and adhesion molecule response to *ex vivo* physiological WSS. **A.** Bar graph of cytokine and adhesion molecule mRNA expression *in vivo*, ex vivo with no WSS, and *ex vivo* with pWSS. No WSS shows an inflammatory response for on P-selectin. Physiological WSS induces an inflammatory response in all except TNFα and VCAM-1. This level of inflammation will be considered as baseline. **B**. Bar graph of growth factor and transcription factor (Egr-1) mRNA expression *in vivo*, *ex vivo* with no WSS, and *ex vivo* with pWSS. Under No Flow conditions no factors are significantly upregulated. Under pWSS, VEGF and Egr-1 show a significant upregulation. Data is normalized to housekeeping gene GAPDH and fold change is expressed with respect to *in vivo* control samples. *P < 0.05, **P < 0.01 *vs*. *in vivo* control samples.

There were dramatic increases in expression of MCP-1, P-selectin and ICAM-1 under No Shear (no WSS) and pWSS (**Fig. 2A**). Specifically, MCP-1 showed an increase of 10.18 ± 3.88 fold (*P* = 0.065) and 11.27 ± 3.94 fold (*P* < 0.05), respectively over *in vivo* values. P-selectin expression increased to 8.34 ± 1.75 fold and 8.25 ± 1.499 fold, respectively (*P* < 0.01 *vs*. *in vivo*). No significant changes were observed when VCAM-1 or TNFα expression was compared for either group.

FGF-2, VEGF and the transcription factor Egr-1 were upregulated under No Shear by 2.06 ± 0.69, 1.5 ± 0.41, and 4.85 ± 1.66 fold respectively but did not reach significances (*P* > 0.05, **Fig. 2B**). FGF-2 was similarly upregulated under pWSS (1.99 ± 0.55 fold). Egr-1 also showed a modest but significant upregulation (2.14 ± 0.4 fold, *P* < 0.05) for pWSS. TGFβ expression did not show any significant differences when each group was analyzed.

### Main femoral artery and branches show no significant differences

Branch mRNA analysis showed similar expression of all markers in each of the three branches with respect to the main femoral artery (**Fig. S3**). For *in vivo*, *ex vivo*, and *ex vivo* no flow, there are no significant differences seen in inflammatory marker expression in any branches (B1, B2, B3) when compared to femoral artery (FA), except with ICAM expression where B1 when No Flow showed modest upregulation (6.11 ± 1.40 fold, *P* < 0.05).

### Inflammatory wall shear stress RNA analysis

**Figure 3.**
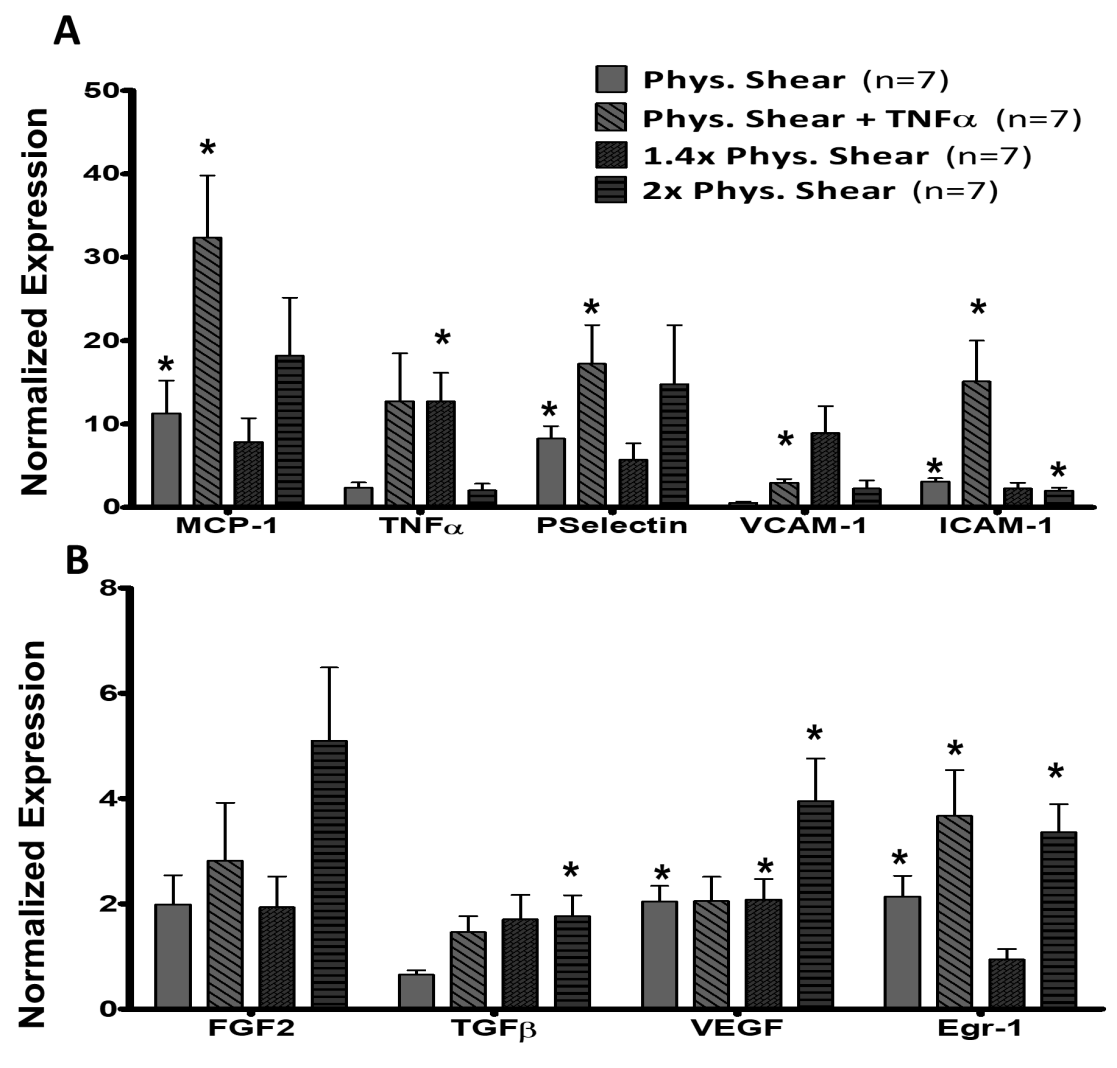
Cytokine and adhesion molecule response to increased *ex vivo* WSS or activation. **A.** Bar graphs representing fold change in mRNA expression of cytokines and adhesions molecules after enduring increased WSS and positive control TNFα stimulation. TNFα shows increases in expression for all genes except TNFα. 1.4× pWSS causes an upregulation of TNFα only. 2× pWSS only shows significant increases for ICAM-1, but trends toward upregulation can be seen in MCP-1 and P-selectin. **B.** Bar graphs representing fold change in mRNA expression of growth factors and transcription factor Egr-1 after enduring increased WSS and positive control TNFα stimulation. 1.4× pWSS only upregulates VEGF, but slightly downregulates Egr-1. 2× pWSS presents with significant upregulation of all factors except FGF-2, even when TNFα did not (FGF-2, TGFβ, VEGF). Data is normalized to housekeeping gene GAPDH and fold change is expressed with respect *in vivo*. *P < 0.05, **P < 0.01 *vs*. *in vivo* control samples.

MCP-1 (*P* < 0.05), P-selectin (*P* < 0.05), VCAM-1 (*P* < 0.05), and ICAM-1 (*P* < 0.05) reached significance in TNFα positive control (**Fig. 3A**) compared to *in vivo* controls. 1.4× and 2× pWSS showed similar expression for MCP-1 (7.86 ± 2.86 and 18.2 ± 6.94 fold respectively), and P-selectin (5.71 ± 1.99 and 14.76 ± 7.1 fold respectively). TNFα expression only reached significance (*P* < 0.01) under 1.4× pWSS (12.72 ± 3.42 fold). ICAM-1 showed similar expression at both 1.4 and 2× pWSS, but only reached significance at 2× pWSS (*P* < 0.05).

TNFα positive control shows an increase in Egr-1 expression (3.67 ± 0.87 fold, *P* < 0.05, **Fig. 3B**). The transcription factor Egr-1 first showed a decrease to 0.94 ± 0.2 fold of control at 1.4× pWSS followed by a significant increase at 2× pWSS of 3.36 ± 0.53 fold (*P* < 0.01). VEGF also shows significant increases at 1.4× and 2× pWSS of 2.08 ± 0.39 and 3.96 ± 0.81 fold, respectively (*P* < 0.05). FGF-2 does not show significant changes expect at 2× pWSS (5.1 ± 1.39, *P* < 0.05). TGFβ did not show any significant changes at any level of shear stress.

### Monocyte adhesion

**Figure 4.**
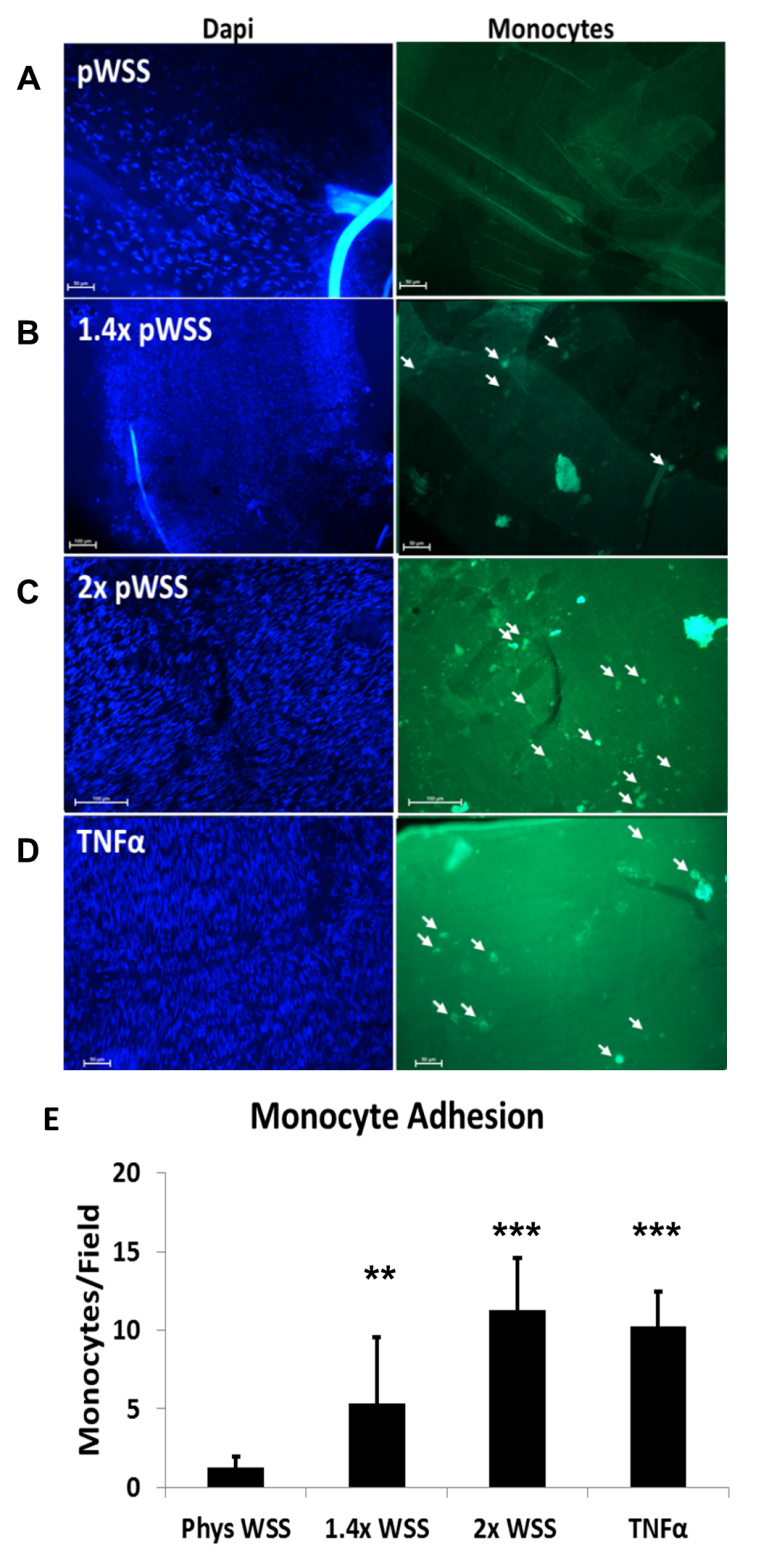
Increased WSS significantly increases static monocyte adhesion. Femoral arteries were perfused at indicated WSS for 24 h. The vessels were then harvested, flayed open and incubated with static, activated, calcein labeled monocytes for 1 h. The vessels were then washed, fixed and mounted with DAPI to visualize the endothelium. Representative images are shown here. **A.** pWSS (*n* = 4): Complete lack of adherent monocytes along the whole femoral artery. **B.** 1.4× pWSS (*n* = 4): Evidence of monocyte adherence along the vessel indicated by the white arrows. **C.** 2× pWSS (*n* = 4): Evidence of increased monocyte adherence along the vessel. **D.** TNFα pWSS (*n* = 4): Evidence of increased monocyte adherence along the vessel. **E.** Graph quantifying the average adherent cells/high powered field. **P < 0.01, ***P < 0.001 *vs*. pWSS.

Quantification of adherent cells on femoral arteries incubated with monocytes compared to pWSS (1.25 ± 0.71 cells/high power field, hpf) and showed an increase in monocyte adherence with TNFα treatment (10.25 ± 2.22 cells/hpf, *P* < 0.001). Significant and WSS dependent increases were also observed at 1.4× pWSS (5.38 ± 4.20 cells/hpf, *P* = 0.01) and 2× pWSS (11.25 ± 3.37 cells/hpf, *P* < 0.001); which represented similar adherence as in TNFα treatment (**Fig. 4**).

## DISCUSSION

The use of an explanted mouse femoral artery and its branches was evaluated for the first time. Many *ex vivo* vessel studies use coronary arteries, aortic arches and rings, thoracic/abdominal aorta, and microvessels (arterioles) [11,13,14,18]. However, *in vivo* occlusion studies are still the most established models for the study of WSS induced inflammation, specifically studies using the FAE or ligation procedures. This *ex vivo* femoral model provides a system through which specific environmental conditions can be altered and investigated while still relating back to a widely accepted *in vivo* models.

### *Ex vivo* femoral artery model

It is not likely that collaterals form from the main femoral artery, but rather off the branches, calling into question the use of full femoral arteries as a model. Here, the use of full femoral arteries is validated quantitatively through the qPCR results showing similar expression of chemo-attractants, adhesion molecules and growth factors between branch and femoral artery expression (**Fig. S3**). This permits the use of the larger and more easily dissected femoral artery for WSS inflammatory studies allowing for larger yields of protein and RNA for analyses.

While many existing models of explanted vessels use similarly controlled flow rate, pressure, temperature; one main variability between studies is the perfusate used. In many studies, perfusate is comprised of basic tissue culture media with specific osmolarity, osmolality and pH. Some systems will add a thickening agent to the media to increase the viscosity to match physiological (40 cP or 4.0 × 10^-3^ Ps) viscosity. In this study, we chose to leave the media less viscous (0.89 cP or 8.9 × 10^-4^ Ps) and increase flow rate to induce physiological shear stress. This was done to mitigate the side effects of using dextran to thicken the media as dextran binds factor VIII-V on Willebrand factor on endothelial cells [19]. The endothelium is the first line of sensing and mechanotransduction of WSS changes as it is responsible for expressing inflammatory cytokines, chemokines, and growth factors in response to inflammatory stimuli [20]. Any binding to the endothelial cells by media additives could be of concern for the expression of adhesion molecules that are critical for expression of the complete inflammatory phenotype [21].

In addition to flow rate, the pulsatility was characterized [22]. The pulsatility is imparted by the roller pump in this study was dependent on the RPM setting. As a result, the pulsatility was not equal to physiological (~630 beats/min), but the goal was to impart the correct maximum shear stress that would be felt by the endothelium *in vivo*. It is admittedly a limitation that the pulsatility cannot be consistent in this model. It should be noted however, that in previous perfusion studies laminar flow has been used as well as roller pumps as in this study [11,13,14,18]. The use of the roller pump was selected because it was deemed important that the vessel experience peak and minimum WSS in both positive and negative directions as occur *in vivo*.

Overall, this study characterized an *ex vivo* femoral artery perfusion system in relation to its use for WSS dependent inflammation models. The data show that the femoral artery’s endothelium and smooth muscle cells are structurally maintained through 24 h of perfusion (**Fig. S2**). Further, this flow is necessary for the vessel to function as close to *in vivo* as possible. While physiological *ex vivo* rates result in an increase in expression of several chemoattractants, adhesion molecules, and growth factors, having no-flow shows an even greater inflammatory response. MCP-1, P-selectin, ICAM-1, VEGF, and Egr-1 were all significantly up-regulated at physiological shear (**Fig. 2**). As long as we assess the baseline inflammatory phenotype after physiological perfusion, this system can be confidently used for inducing specific peak WSS, and identifying chemo-attractants, adhesion molecules, and growth factors indicating inflammation. We believe the differential expression of these and other inflammatory response genes with increased *ex vivo* shear stress overcome this limitation and make this model useful in gauging the effect of increased shear stress. However, it is advisable to perform *in vivo* experiments to substantiate the results of this model.

### Increased WSS

In this study, two increased WSS conditions were analyzed in respect to the baseline of pWSS with TNFα positive control as a reference. We establish here that increase in WSS results in greater expression patterns that are discernable beyond the established baseline (**Fig. 3A** and **3B**). TNFα and ICAM-1 were significantly upregulated while MCP-1, VCAM-1, and P-selectin showed a strong trend toward upregulation with increases in WSS. These same markers are upregulated in *in vivo* occlusion studies [23,24].

We further hypothesized that there would not be an upregulation of growth factors from an increase in shear stress alone. It was expected that the recruitment and influx of leukocytes would be necessary to see any viable increases in growth factor expression [25]. However, results show that the selected growth factors do in fact increase in expression with the increases in WSS alone (**Fig. 3B**).

One possible explanation is that Egr-1 is the driving force regulating vessel response to increased WSS. In both 2× pWSS and TNFα positive control, Egr-1 is upregulated from pWSS. It is known that Egr-1 is a transcriptional factor that plays a role in both pushing the vascular endothelium and VSMCs towards a synthetic phenotype. It is possible that Egr-1 is triggered immediately by the increase in WSS or other inflammatory activity (such as TNFα upregulation), which then causes a signaling cascade to initiate the inflammatory and synthetic phenotype for the endothelial cells. This response progresses through to the VSMC which themselves enter into a synthetic phenotype.

This leads into the question of why does 1.4× pWSS cause a downregulation of Egr-1. While we were expecting a “dose-response” to WSS, it is possible that there is a threshold that needs to be achieved in order for Egr-1 to initiate. While a modest inflammatory response is elicited, as perhaps indicated by the expression of TNFα and VEGF, a full response is predicated on a specific threshold to be met. The nature of the pulsatility of the vessel perfusion should also be considered. The pulsatility at 1.4× pWSS is a lower frequency than at 2× pWSS. Perhaps this plays into the mechanotransduction and signal sensing that the max WSS is being applied at a greater rate at 2× pWSS than at 1.4× pWSS.

Egr-1 has been shown in recent literature to be a potential “master switch” in arteriogenesis [24,26]. Knockout studies have shown that without Egr-1 the arteriogenic response following occlusion is severely blocked [24]. Preliminary studies in our lab have also shown that inflammatory response and monocyte recruitment is diminished in the case of Egr-1 knockouts after femoral artery excision (data not shown). Further studies using this femoral artery model with Egr-1 knockout arteries could provide valuable information regarding the mechanistic role of Egr-1 in initiating arteriogenic growth.

We can conclude from this study that WSS has a discrete role in inducing an inflammatory phenotype; triggering the expression of cytokines, adhesion molecules and growth factors. Further, this *ex vivo* femoral artery model is a viable platform for analyzing these changes in a simplified setting that is more easily accessed and manipulated. Following studies addressing limitations due to perfusion methods (pulsatility), as well as those assessing multiple time points and more levels of WSS, this model should be able to assess the mechanisms and time frame of the arteriogenic response with respect to WSS.

The static monocyte incubation on arteries with the inflammatory phenotype induced by WSS shows that the inflammatory phenotype has direct implications on monocyte adhesion. Specifically, higher WSS means more adhesion molecules and subsequent monocyte adhesion. Previous studies have done this type of static monocyte incubation on various activated endothelial cells [27]. Additionally, the 2× pWSS showed a more robust inflammatory and growth factor mRNA expression response than 1.4×. This is in line with the increased monocyte adhesion we see after static incubation. In addition to monocyte adhesion, this shows us that the inflammatory phenotype is sustained even after removing the vessel from perfusion, for at least 1 h. Future efforts to show real-time recruitment of monocyte adhesion may be possible with reduced flow rate.

## Supplementary Material

Supplementary information **Figure S1**. Schematics showing the *ex vivo* set up for femoral artery perfusion.
**Figure S2**. Femoral arteries were perfused for 24 h and stained to visualize vessel structure.
**Figure S3**. Bar graphs reresenting mRNA expression of MCP-1, P-selectin, VCAM-1 and ICAM-1 in the three branches of the femoral artery, with respect to the main femoral artery.
Supplementary information of this article can be found online at
http://www.jbmethods.org/jbm/rt/suppFiles/274.

## Figures and Tables

**Table 1.** Descriptions of flow rate, RPM, WSS, media, and maximum pressure for each of the WSS treatments tested.

	pWSS	1.4× pWSS	2× pWSS	TNFα control
Flow rate (ml/min)	0.4319	0.5682	0.7727	0.4319
RPM	21.5	27.8	37.1	21.5
WSS	10.18 Pa	14.26 Pa	20.37 Pa	10.18
Media	DMEM + 10% FBS	DMEM + 10% FBS	DMEM + 10% FBS	DMEM + 10% FBS + 1 μg/ml TNFα
Max. pressure (mmHg)	80	80	80	80

**Table 2.** Comparison of *in vivo* and *ex vivo* arterial parameters.

C57BL/6 mouse femoral artery	*In vivo*	*Ex vivo*
Peak wall shear stress (*τ*_ω_; Pa)	10.18	10.18
Internal radius (*D*; mm)	0.20	0.20
Viscosity (*η*; Pa·s)	*η*_blood_ = 4.0 × 10^-3^	*η*_DMEM_ = 8.9 × 10^-4^
Peak flow rate (*Q*; ml/min)	*Q*_invivo_ = 0.12	*Q*_exvivo_ = 0.53
